# Recombinant expression of *Chlamydia trachomatis* major outer membrane protein in *E. Coli* outer membrane as a substrate for vaccine research

**DOI:** 10.1186/s12866-016-0787-3

**Published:** 2016-07-27

**Authors:** Zhiyun Wen, Melissa A. Boddicker, Robin M. Kaufhold, Puneet Khandelwal, Eberhard Durr, Ping Qiu, Bob J. Lucas, Debbie D. Nahas, James C. Cook, Sinoeun Touch, Julie M. Skinner, Amy S. Espeseth, Craig T. Przysiecki, Lan Zhang

**Affiliations:** 1 Infectious Diseases and Vaccines Discovery (West Point, PA), MRL, Merck & Co., Inc, Kenilworth, NJ USA; 2 Translational Molecular Biomarkers (Rahway, NJ), MRL, Merck & Co., Inc, Kenilworth, NJ USA

**Keywords:** *Chlamydia trachomatis*, MOMP, Outer membrane expression, Immunogenicity

## Abstract

**Background:**

*Chlamydia trachomatis* is a human pathogen which causes a number of pathologies, including genital tract infections in women that can result in tubal infertility. Prevention of infection and disease control might be achieved through vaccination; however, a safe, efficacious and cost-effective vaccine against *C. trachomatis* infection remains an unmet medical need. *C. trachomatis* major outer membrane protein (MOMP), a β-barrel integral outer membrane protein, is the most abundant antigen in the outer membrane of the bacterium and has been evaluated as a subunit vaccine candidate. Recombinant MOMP (rMOMP) expressed in *E. coli* cytoplasm forms inclusion bodies and rMOMP extracted from inclusion bodies results in a reduced level of protection compared to the native MOMP in a mouse challenge model.

**Results:**

We sought to target the recombinant expression of MOMP to the *E. coli* outer membrane (OM). Successful surface expression was achieved with codon harmonization, utilization of low copy number vectors and promoters with moderate strength, suitable leader sequences and optimization of cell culture conditions. rMOMP was extracted from *E. coli* outer membrane, purified, and characterized biophysically. The OM expressed and purified rMOMP is immunogenic in mice and elicits antibodies that react to the native antigen, *Chlamydia* elementary body (EB).

**Conclusions:**

*C. trachomatis* MOMP was functionally expressed on the surface of *E. coli* outer membrane. The OM expressed and purified rMOMP elicits antibodies that react to the native antigen, *Chlamydia* EB, in a mouse immunogenicity model. Surface expression of MOMP could provide useful reagents for vaccine research, and the methodology could serve as a platform to produce other outer membrane proteins recombinantly.

## Background

*Chlamydia trachomatis* is an obligate intracellular Gram-negative bacterium responsible for a number of pathologies. Different strains of *C. trachomatis* are separated into multiple serovars based on serological differences in the *Chlamydia* major outer membrane protein (MOMP) [1, 2]. *C. trachomatis* serovars A, B, Ba, and C are responsible for ocular trachoma which can cause conjunctivitis, conjunctival scarring and corneal scarring. Serovars L1, L2 and L3 are responsible for lymphogranuloma venereum, which can cause submucosa and lymph-node invasion, with necrotizing granulomas and fibrosis. Serovars D, Da, E, F, G, H, I, Ia, J, Ja and K are responsible for oculogenital disease which can cause cervicitis, urethritis, endometritis, pelvic inflammatory disease, tubal infertility, ectopic pregnancy, neonatal conjunctivitis and infant pneumonia [3, 4]. *C. trachomatis* infection is one of the most common sexually transmitted diseases in the world and imposes a significant medical and economic burden [5–7]. Therefore, a prophylactic vaccine is desirable to prevent *C. trachomatis* related diseases.

Past vaccine candidates comprised of formalin-inactivated *Chlamydia trachomatis* were only able to elicit short lived protection [8]. The subunit vaccine approach has been investigated as an alternative. The *Chlamydia* bacterium exists outside the host cell as a metabolically inactive infectious elementary body (EB) and the *Chlamydia* MOMP accounts for about 60 % of the mass of the outer membrane of EBs [9, 10]. Antibodies against MOMP can neutralize infectivity of EBs and T cell epitopes have also been identified in MOMP [11, 12]. Therefore, MOMP has been an attractive subunit vaccine candidate against *Chlamydia* infection for many researchers [13–18]. Native MOMP (nMOMP) antigen isolated from EBs induced a protective immune response to a *C. trachomatis* genital challenge infection in a mouse model [14, 15]. However, development of a robust, cost-effective commercial manufacturing process based on the use of native MOMP can be challenging. Recombinant expression of a vaccine antigen is an alternative method to purification of native antigen from infected cells, and may be easier to scale-up to a commercial manufacturing level.

*Chlamydia* MOMPs are part of a larger family of genetically related outer membrane proteins (the OmpA family) that are heat-modifiable, surface exposed porin proteins [19]. OmpA proteins have a structurally similar N-terminal domain that is embedded in the bacterial outer membrane. OmpA proteins have been targeted as vaccine candidates because of their surface exposure, high immunogenicity, and role in the interaction between the bacteria and their host cells. Unlike most outer membrane β-barrel proteins, MOMP is cysteine rich and was predicted to be a 16 stranded β-barrel outer membrane protein [20, 21]. Recombinantly expressed MOMP (rMOMP) has been explored as a vaccine candidate. rMOMP expressed without a leader sequence forms inclusion bodies in the cytoplasm of *E. coli* and rMOMP extracted from inclusion bodies resulted in a reduced level of protection compared to the native MOMP in a mouse challenge model [22, 23]. This may be due to the extracted and refolded rMOMP not having the correct conformation. Thus as an alternative, an outer membrane (OM) expressed rMOMP may attain the correct conformation and enhance the vaccine efficacy. Previous OM expression of rMOMP in *E. coli* resulted in significant reduction in cell viability [22, 24]. Findley *et al.* has shown improved surface expression of rMOMP, however did not report immunogenicity of the expressed OM rMOMP in an animal model [25]. In this study, expression of rMOMP in *E. coli* outer membrane was optimized by using a number of strategies including rMOMP gene codon harmonization, utilization of low copy number vectors and promoters with moderate strength, evaluation of leader sequences and optimization of cell culture conditions. rMOMP was extracted, purified and biophysically characterized. We show here that *E. coli* OM expressed rMOMP possessed β-barrel structure and elicits serum antibodies that react to the native antigen, *Chlamydia* elementary body, in a mouse immunogenicity model.

## Methods

### Codon harmonization of the *Chlamydia* MOMP gene for recombinant expression

Nucleotide sequences of the gene encoding MOMP were retrieved from Merck internal website CMR (Comprehensive Microbial Resources) for the following strains: *C. muridarum* Nigg (strain MoPn) ORF TC0052 (GenBank Gene ID: 1245581; Protein link P75024.1); *C. trachomatis* strain D/UW-3/CX CT ORF TC_681 Serovar D (GenBank Gene ID: 884473; Protein link NP_220200.1); and *C. trachomatis* strain E/12–94 ORF O175_03780 Serovar E (GenBank Gene ID: 16635280; Protein link P17451). Amino acid sequences consisting of a secretion leader and the mature MOMP protein (Table 1) were codon harmonized [26, 27]. In brief, the codon usage data for *Chlamydia* (native host) and *E. coli* (expression host) were obtained from the Codon Usage Database *(*http://www.kazusa.or.jp/codon/*)*. For each species, the strain with the most codon usage data available was selected as a representative. The codon usage frequency for both native and expression hosts was then calculated and a reference database was generated. We first identified the amino acid residues for which the rare codons were used in the native host, and the corresponding rare codon in expression host was selected for those residues. For the remaining residues, the codon in the expression host that has the closest frequency to the corresponding codon in the native host was selected, and the harmonized gene sequence for *Chlamydia* MOMP was then generated. NdeI and XhoI sites were avoided in the sequences for subsequent cloning.

**Table 1:** Evaluation of Secretion Leader Sequences

Secretion Leader	Amino Acid Sequences	rMOMP Surface Expression		
		Cm-MOMP	CtD-MOMP	CtE-MOMP
Native Cm-MOMP	MKKLLKSVLAFAVLGSASSLHA	+	-	-
Native CtD/CtE-MOMP	MKKLLKSVLVFAALGSASSLQA	ND	-	-
*Shigella flexneri* (SopA)	MKSKFLVLALCVPAIFTTHA	ND	ND	+
*Salmonella enterica* (PgtE)	MKTHVIAVMIIAVFSESVYA	ND	ND	+
*Yersinia pestis* (Pla)	MKKSSIVATIITILSGSANA	ND	ND	+
*E. coli* OmpP	MQTKLLAIMLAAPVVFSSQEASA	ND	ND	+
*E. coli* OmpA	MKKTAIAIAVALAGFATVAQA	+	ND	+
pectate lyase B of *Erwinia carotovora* CE (PelB)	MKYLLPTAAAGLLLLAAQPAMA	+	+	+

*ND:* Not Determined

### Cloning and expression of recombinant *Chlamydia* MOMP

The harmonized gene sequences with flanking NdeI and XhoI restriction enzyme sites were synthesized and cloned into the pUC57 cloning vector (Genewiz). The synthesized genes were excised from pUC57 vector through NdeI and XhoI resitriction sites. The excised DNA fragments were ligated into the pAVE029 expression vector (MSD Biologics UK) using T4 DNA ligase (Promega) for 4 h at 16 °C. Ligated plasmids were transformed into competent cells DH5α (Invitrogen) and grown on LB agar plates with 10 μg/mL tetracycline. Colonies harboring the recombinant plasmid were identified by PCR and confirmed by sequencing using pAVE029 vector specific primers for 5′end of the gene (ppop40 primer ATTCTGCATTCACTGGCCGAGG) and 3′end of the gene (T7 Term standard sequencing primer GCTAGTTATTGCTCAGCGG). The sequence-confirmed positive colonies were propagated in LB medium with 10 μg/mL of tetracycline and plasmid DNA was isolated from the cell cultures with HiSpeed Maxi Kit (Qiagen).

The recombinant plasmid DNA was transformed by electroporation into an expression host strain *E. coli* K12 W25113 using a Bio-Rad GenePulser. Transformed cells were plated on LB Agar plates with 10 μg/mL tetracycline and grown overnight at 37 °C. Single colonies were picked and inoculated into Cinnabar media (Teknova) with 10 μg/mL of tetracycline and grown at 37 °C with shaking at 250 rpm until OD_600_ reaches to mid log phase (~0.5). 0.4 mM IPTG was added into the cell culture for induction and the cell culture was incubated for 4 h at 30 °C with shaking. The cell cultures were then characterized by whole cell flow cytometry antibody binding, SDS-PAGE, and western blot analyses.

### Whole cell flow cytometry antibody binding

50 μL of *E. coli* cell culture (at ~1x10^9^ cells/mL) that recombinantly expresses *Chlamydia* MOMP was incubated with 50 μL of mouse sera generated in-house against *Chlamydia* EBs at a dilution of 1:250 for 1 h at room temperature in a 96 well plate. After incubation, the cells were washed with 1 mL phosphate buffered saline (PBS) and stained with 100 μL of a fluorescence labeled secondary antibody (Alexa Fluor-488 F(ab)’2 fragment of goat anti-mouse IgG (H + L), Life Technologies) at a dilution of 1:100. The stained cells were washed twice and re-suspended in PBS for flow cytometric analysis (Guava Technologies). Data analyses were performed with CytoSoft 5.3 software (Guava Technologies).

### SDS-PAGE and western blot

*E. coli* cell culture (~1x10^9^ cells) that recombinantly expresses *Chlamydia* MOMP was treated with SDS loading buffer with reducing agent (Invitrogen). Samples were applied to NuPAGE (Invitrogen) gel electrophoresis. NuPAGE gel was stained with Gel Code Blue staining solution (Pierce). For western blot, samples were applied to gel electrophoresis and then electro-transferred onto nitrocellulose membranes (Life Technologies). The membranes were incubated with mouse sera against *Chlamydia* EBs (or other specific primary antibodies) followed by a fluorescence conjugated goat anti-mouse secondary antibody (IRDye 680LT, Licor). Image was acquired and analyzed by a LI-COR Odyssey imaging system.

### Purification of *Chlamydia* MOMP

*E. coli* cell culture was grown in Cinnabar media (Teknova) and induced by IPTG as described above. Cell culture was harvested by centrifugation at 12,000 x g for 15 min. Cell pellets were weighed and resuspended in 9 volumes (v/w) of 50 mM Tris-Cl pH 8.0 buffer with EDTA free protease inhibitor (Roche, 1 tab per 100 mL buffer). Cells were disrupted by microfluidization and undisrupted cells were pelleted and removed by centrifugation at 9700 x g for 15 min. Membrane fraction was pelleted by centrifugation of the cleared disrupted cells at 23800 x g for 90 min and washed with high salt buffer (1 M NaCl, 0.05 % tween20) followed by another centrifugation at 23800 x g for 90 min. To remove the bacterial inner membrane, washed membrane fraction was resuspended in buffer A (20 mM Tris-Cl pH 8.0, 1 mM EDTA) with 1 % Triton X-100, incubated at room temperature for 15 min followed by ultracentrifugation at 120,000 x g for 40 min. To remove bacterial outer membrane proteins other than recombinant MOMP, pellets were resuspended by buffer A with 3 % β-octyl-glucoside, incubated at room temperature for 1 h followed by ultracentrifugation at 120,000 x g for 40 min. rMOMP was extracted by resuspending the pellets in buffer A with 1 % sarkosyl and 5 mM DTT, incubated at room temperature for 2 h followed by ultracentrifugation at 120,000 x g for 40 min. Extracted rMOMP was subjected to size exclusion chromatography (Sephacryl S300, GE healthcare) in a buffer containing 10 mM Hepes pH 7.3, 150 mM NaCl, 0.1 % zwittergent 3–14. Eluted rMOMP was further purified with ion exchange chromatography (Hitrap Q FF, GE healthcare). Purified rMOMP fractions were pooled and stored at 4 °C. Native MOMP was purified from infected HELA cells similarly as previously described [28]. Briefly, nMOMP was extracted from intact *Chlamydia*-infected HELA cells using CHAPS and zwittergent 3–14 with 100 mM DTT and sonication, and further purified with hydroxyapatite chromatography, tangential flow filtration, and anion exchange chromatography.

### Circular dichroism spectroscopy

Circular dichroism spectra were acquired on a Chirascan spectrometer (Applied Photophysics LtD, UK). Samples were analyzed undiluted using a quartz cuvette with 0.5 mm pathlength. The temperature control was set to 20 °C. The bandwidth was set to 1 nm and data points between 200 nm and 280 nm were acquired in 1 nm intervals. Scans below 200 nm exceeded the allowable photomultiplier voltage and were not recorded. Sample and buffer spectra were acquired after 10 min of temperature equilibration applying three technical replicates, respectively. Average buffer spectra were subtracted from sample spectra. Resulting data points were smoothed with the Savitzky-Golay algorithm (polynomial order 2, two data points to left and right) using the Origin Pro 7.5 SR7 software package (Origin Lab Corporation).

### Cell culture and propagation of *Chlamydiae*

All cell lines and *Chlamydia* strains were obtained from ATCC (Manassas, VA). HeLa 229 cells were used for propagation of all strains. HeLa 229 cells were grown in Eagle’s Minimal Essential Medium (ATCC) supplemented with 10 % heat-inactivated fetal bovine serum (Hyclone), 50 μg/mL vancomycin (Sigma), and 10 μg/mL gentamicin (Gibco). Host cells were seeded into tissue culture flasks at a cell density of 5 × 10^5^ cells/mL and incubated overnight at 37 °C in 5 % CO_2_ to achieve a confluent monolayer. Cell monolayers were infected with *C. trachomatis* (Ct) strain D/UW-2/Cx stock diluted in sucrose–phosphate–glutamate (SPG) buffer and cultured for 72 h. The *Chlamydiae* were harvested from the infected cells and purified by centrifugation through 30 % Renograffin (Bracco Diagnostics) and stored frozen at -80 °C.

### Mouse immunization and challenge

Female C57BL/6 mice (Taconic Farms) were used at 6 to 8 weeks of age, and food and water were provided ad libitum. All animal procedures were in accordance with government and institutional guidelines for animal health and well-being, and were approved by the Merck Institutional Animal Care and Use Committee.

Animals were immunized by subcutaneous (s.c.) routes with rMOMP or nMOMP (10 μg/mouse/immunization) in combination with an adjuvant containing 50 μg CpG and 70 % (w/v) Montanide ISA 720 VG (SEPPIC Inc., Coley Pharmaceutical Group). Live EB groups were immunized with 1x10^6^ EB in SPG per mouse by intraperitoneal (i.p.) route. Adjuvant control groups were administered with a combination of CpG and Montanide ISA 720 VG only. Immunizations were administered on days 0, 20 and 30.

Prior to the first immunization and two weeks following the final immunization, tail bleeds were performed with blood collected in BD Microtainer® Serum Separator Tubes (Becton, Dickinson and Company). Blood samples were centrifuged at 6,000 rpm for 5 min and serum was transferred to a microcentrifuge tube.

At approximately 2 weeks following the last immunization, progesterone (medroxyprogesterone acetate, Depo-Provera; Pfizer) was administered subcutaneously (2.5 mg/dose) at 10 and 3 days before challenge. Mice were challenged intravaginally (approximately 1 month following the last immunization) by direct instillation of 10 μL of SPG containing 1x10^5^ Ct serovar D EBs. The vaginal vault and ectocervix were swabbed using a microfiber swab (Fisher) on days 7, 11, 14, 18, and 21 (or a combination of these time points) following challenge.

Swabs were placed into a 1.5-mL tube containing 2 sterile glass beads (5 mm diameter) and 300 μL of *Chlamydia* isolation medium (Trinity Biotech) on ice. Bacteria were eluted from the swabs and separated from cells by vortexing for 60 s. 100 μL of eluted cells/bacteria were plated onto a processing cartridge containing 100 μL of PBS and stored at -70 °C until DNA extraction.

### Primer, probe and real-time PCR

DNA from genital swab samples was extracted using the MagNA Pure 96 DNA and Viral NA small volume kit (Roche) on the MagNA pure machine (Roche) according to the manufacturer’s instructions.

The oligonucleotide primer set was designed for detection of all species of *Chlamydiae*. The sense primer, 16S DIR 5′-CGCCTGAGGAGTACACTCGC-3′, and anti-sense primer, 16S Rev 5′-CCAACACCTCACGGCACGAG-3′, were designed to amplify a 208-bp fragment of the *Chlamydial* 16S ribosomal subunit gene, conserved across *Chlamydia* strains and serovars. Primers were obtained from Sigma Genosys (The Woodlands, TX), and the probe, 16S Fam-5′-CACAAGCAGTGGAGCATGTGGTTTAA-3′ Tamra, was synthesized by Applied Biosystems, (Foster City, CA).

The 50-μL reaction mixtures consisted of 1× QuantiTect Multiplex PCR master mix without ROX (Qiagen), 100 nmol/L 16S probe, 200 nmol/L primer 16S DIR, 400 nmol/L primer 16S Rev, 30 nmol/L ROX reference dye, and 5 μL of sample DNA. Nontemplate controls consisting of the reaction master mix, primers, and probe, but no DNA, were included in each assay run. Reaction conditions were set as follows: 1 cycle at 95 °C for 15 min, followed by 40 cycles at 94 °C for 1 min and at 60 °C for 1 min. Thermal cycling, fluorescent data collection, and data analysis were performed using the Stratagene Mx3005P system (Stratagene) according to the manufacturer’s instructions.

### Detection of serum antibody and isotype levels by ELISA

Serum was analyzed by an enzyme-linked immunosorbent assay (ELISA). Nunc ™ C96 Maxisorp Immunoplates (Thermo Scientific) were coated with 50 μL of 1 μg/mL *C. trachomatis* Serovar D EBs in PBS and refrigerated overnight. The plates were washed three times with 0.05 % Tween-20 (Fisher Scientific) in PBS (PBS-T). The wells were blocked with 5 % HyClone® fetal bovine serum (Thermo Scientific) in PBS at 200 μL/well for 1 h at room temperature and washed three times with PBS-T. Serum was diluted in 5 % FBS in PBS at a 1:500 dilution. Serially diluted sera were added to the plate, incubated for 2 h at room temperature and the plates were washed three times with PBS-T. HRP-conjugated secondary antibodies (Goat anti-mouse IgG, Fcγ fragment specific; Goat Anti-mouse IgG, Fcγ Subclass 1 specific; or Goat Anti-mouse IgG, Fcγ Subclass 2c specific; Jackson ImmunoResearch Laboratories) were diluted in 5 % FBS in PBS at 1:6,000, 1:6,000, or 1:2,000 dilution, respectively. The diluted secondary antibodies were added at 100 μL/well, incubated for 1 h at room temperature and the plates were washed three times with PBS-T followed by three times with PBS. Room temperature BD Opt EIA™ TMB Substrate Reagent Set (BD Biosciences) was mixed and filtered through a 0.22um CA filter unit (Corning), and 100 μL was added to each well and incubated for 10 min at room temperature. The reaction was stopped with 100 μL/well of 2 M H_2_SO_4_ (Fisher Scientific). The optical density (OD) was read at 450 nm on a SpectraMax® M5 (Molecular Devices). The cutoff OD for each post-immunization serum was calculated as two times of the OD_450_ of the corresponding pre-immunization serum. ELISA titers were determined by linearly interpolating between the sequential log dilutions that bracket the cutoff OD, where the dependent variable is the OD response and the independent variable is the log dilution. The resulting dilution is then back transformed to obtain the reported titer. The reported titer is the estimated dilution of serum that results in a response equivalent to the cutoff OD.

## Results

### Expression of recombinant *Chlamydia* MOMP

#### Codon harmonization

**Fig. 1:**
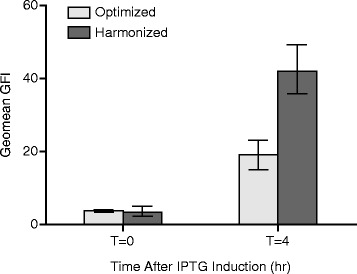
Effect of codon usage harmonization on rMOMP surface expression level. FACS geometric mean (geomean) fluorescence intensity (GFI) is shown for harmonized or optimized rCm-MOMP genes at T = 0 and T = 4 h after IPTG induction. Geometric means of GFI from three independent experiments were plotted with error bars representing standard deviation

To improve recombinant protein expression in a heterologous host, Angov *et al.* developed an algorithm termed “codon harmonization” that best approximates codon usage frequencies from the native host and adjusts these for use in the heterologous system [26, 27]. We performed either codon harmonization or standard codon optimization on a recombinant *Chlamydia muridarum* (Cm) MOMP gene and evaluated outer membrane expression with a whole cell flow cytometry antibody binding assay using in-house generated polyclonal mouse sera against *Chlamydia* EB. Both codon type genes were expressed in a pET vector expression system and with a MOMP native leader sequence. We observed that codon harmonization resulted in ~2 fold increase in geomean fluorescence intensity (GFI) compared to the standard host codon optimized gene (Fig. 1). Therefore, codon harmonized genes were used in subsequent expression evaluations (data below) to further improve rMOMP OM expression.

#### Expression vector optimization

A panel of *E. coli* expression vectors were evaluated to further increase the surface expression level of rMOMP (Table 2). Recombinant *C. trachomatis* Serovar E (CtE) MOMP with a PelB leader sequence was used in this evaluation. The key elements that could affect the OM expression include promoter strength and vector copy number. We compared vectors with high, medium or low copy numbers, and promoters of high, moderate or titratable strength. We found that either a strong promoter or a high vector copy number limited the surface expression of rMOMP using whole cell flow cytometry antibody binding assay (Table 2). High rMOMP surface expression can only be achieved with a combination of moderate promoter and a low vector copy number (such as pAVE029), suggesting that lower transcription level is preferred. Consistent, rMOMP OM expression can be obtained with a pACYDuet vector when we used a host strain with a controllable RNA polymerase level to reduce the rMOMP mRNA transcription rate.

**Table 2:** Evaluation of *E. coli* expression vectors

Vector	Promoter/Strength	Inducer	Origin of Replication	Copy Number	rCtE-MOMP Surface Expression (GFI^a^)
^b^pAVE029	λPL/Moderate	IPTG	pAT153(colE1)	Low	Good (~300)
pACYDuet-1	T7/Titratable	Arabinose + IPTG	p15A	Low	Intermediate (~120)
pET (pETBlue-1 and pET22b)	T7/Strong	IPTG	pUC	High	Low (~30 or lower)
pWSK29	T7/T3/Strong	IPTG	pSC101	Low	None (intracellular)
pJ831(pUC)	T7/Titratable	Rhamnose	pMB1	High	None (intracellular)
pJ841(pBR)	T7/Titratable	Rhamnose	pMB1	Medium	None (intracellular)
pJ851(pACYC)	T7/Titratable	Rhamnose	p15A	Low	None (intracellular)

^a^GFI: geomean fluorescence intensity from whole cell flow cytometry antibody binding assay

^b^pAVE029 is an *E. coli* RNA polymerase dependent expression vector. Others listed are bacteriophage T7 RNA polymerase dependent expression vectors

#### Secretion leader sequence optimization

There are several advantages of secretory production of recombinant membrane proteins compared to cytosolic production. The N-terminal amino acid residue of the secreted product can be identical to the natural gene product after cleavage of the signal sequence by a specific signal peptidase. Also, there appears to be much less protease activity in the periplasmic space than in the cytoplasm [29]. In addition, disulfide bonds formation can be facilitated because the periplasmic space provides a more oxidative environment than the cytoplasm [29]. Enhanced OM insertion and prevention of periplasmic space inclusion bodies were investigated. Two amino acids at the C-terminus of CtD-MOMP (*C. trachomatis* Serovar D) were modified to improve the binding to the *E. coli* β-barrel assembly complex protein (BamA), which recognizes its outer membrane protein substrates by a species-specific C-terminal signature motif [30]. However, we did not observe a difference in surface expression levels between the wild type CtD-MOMP and the C-terminus modified CtD-MOMP sequences (unpublished observations). Different secretion leader sequences reported to improve the OM localization of the target protein were investigated by using a whole cell flow cytometry antibody binding assay (Table 1). pAVE029 expression vector was used in this evaluation for Cm-MOMP, CtE-MOMP and CtD-MOMP. Among tested leader sequences, *E. coli* OmpA and OmpP leaders, leader sequences from the omptins (SopA, PgtE, and Pla), and PelB leader all resulted in detectable surface expression of rMOMP. Native Cm-MOMP leader was able to direct the OM expression for Cm-MOMP, however, neither of native CtD- or CtE-MOMP leaders resulted in the surface expression of rMOMP.

#### Expression condition optimization

**Fig. 2:**
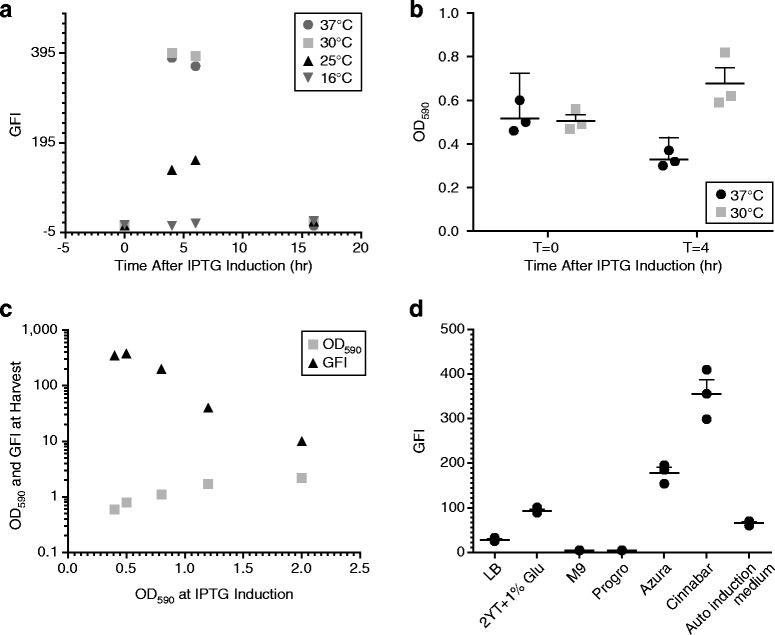
Evaluation of expression conditions. rCtE-MOMP with a PelB leader sequence constructed in the pAVE029 vector was evaluated. **a** Surface expression (FACS GFI) of rCtE-MOMP after different IPTG induction times (T = 0 and T = 4 h) when expressed at different temperatures (37 °C = circles, 30 °C = squares, 25 °C = triangles, 16 °C = downward pointing triangles). **b** Cell concentration (OD_590_) at different temperatures (37 °C = circles, 30 °C = squares) after different IPTG induction times (T = 0 and T = 4 h). Geometric means of three independent experiments were plotted with error bars representing standard deviation. **c** Cell concentration (OD_590_) and surface expression (FACS GFI) at harvest with different cell densities (OD_590_) at IPTG induction (T = 0). **d** Surface expression (FACS GFI) with different cell culture media. Geometric means of replicates were plotted with error bars representing standard deviation

Surface expression of rMOMP has been associated with toxicity to the host cell, resulting in a low yield [22, 24, 25]. We evaluated a variety of cell culture conditions with the pAVE029 expression system, including induction time and temperature, cell density at induction, and cell culture medium (Fig. 2). rCtE-MOMP with a PelB leader sequence was used in this evaluation. We performed induction for 4 h, 6 h and 16 h under four different temperatures: 16 °C, 25 °C, 30 °C and 37 °C. We found that 4 h and 6 h resulted in comparable rMOMP OM expression levels while no expression was observed following 16 h induction at any of the temperatures tested (Fig. 2a). Induction for 4 or 6 h at 37 °C or 30 °C resulted in higher surface expression of rMOMP than 25 °C and no detectable surface expression was observed at 16 °C (Fig. 2a). We also observed cell fragility at 37 °C (but not at 30 °C), which was indicated by a decrease in OD_590_ after induction (Fig. 2b). We performed induction at different cell densities and found that it dramatically impacts the rMOMP surface expression. The highest rMOMP expression was obtained with an induction OD_590_ of ~0.5, while expression dropped with an induction OD_590_ of ~0.8, while little or no surface expression of rMOMP was observed with an induction OD_590_ of 1.2 or higher (Fig. 2c). We evaluated seven cell culture mediums and interestingly, the culture medium used had a large impact on rMOMP OM expression (Fig. 2d). Low levels of rMOMP OM expression were observed with LB medium, 0.2 % lactose auto induction medium, 2YT medium with 1 % glucose, and the chemically defined Azura medium. The highest rMOMP OM expression (GFI ~300 to 400 in the whole cell flow cytometry antibody binding assay) was obtained with growth in Cinnabar medium. We obtained very high level of periplasmic rMOMP expression with Progro medium, however, no surface expression was observed. IPTG concentration for induction was also evaluated and comparable rMOMP OM expression was observed with 0.1 mM to 1 mM IPTG (unpublished observations). Therefore, all expression studies described here were induced by addition of 0.4 mM IPTG.

**Fig. 3:**
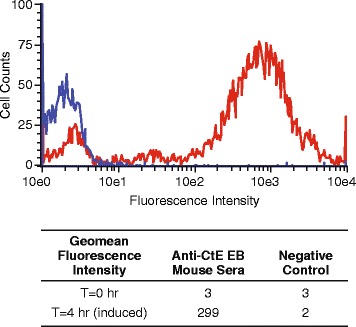
Expression of rCtE-MOMP on *E. coli* outer membrane under optimized expression conditions. An *E. coli* transformant expressing rCtE-MOMP with a PelB leader constructed in a pAVE029 vector was grown in Cinnabar medium at 37 °C and induced by 1 mM IPTG at 30 °C for 4 h when OD_590_ reaches ~0.5. Whole cell flow cytometry antibody binding histograms were shown: red, anti-CtE EB mouse sera and blue, a negative control antibody. Geomean fluorescence intensities were also shown in table

In summary, optimal conditions for rCtE-MOMP OM expression was found to be induction for 4 h at 30 °C in cells grown in Cinnabar medium when cell density (OD_590_) reaches ~0.5 (Fig. 3). Three different rMOMP proteins (Cm, CtD and CtE) have been successfully expressed on *E. coli* outer membrane under these conditions. rCtE-MOMP with a PelB leader sequence was used for the subsequent purification, characterization and immunogenicity studies in this manuscript.

### Purification and characterization of recombinant *Chlamydia* MOMP

Following optimization of rCtE-MOMP OM expression, the recombinant protein was purified. Harvested *E. coli* cells expressing rMOMP were disrupted by microfludization and the membrane fraction containing rMOMP was pelleted by ultra-centrifugation. Washing the membrane fraction with high salt buffer further removed residual soluble cellular proteins. A subsequent wash with a buffer containing 1 % Triton X-100 detergent removed the bacterial inner membrane and a wash with a buffer containing 3 % β-octyl-glucoside detergent removed certain bacterial outer membrane proteins other than recombinant MOMP. A variety of detergents were evaluated for extraction of rMOMP from the outer membrane. We found that sarkosyl (an anionic detergent) was the most efficient, followed by foscholine-14 (a lipid like zwitterionic detergent) and zwittergent 3–12. DTT was required for extraction of rMOMP. Sarkosyl extracted rMOMP was further purified by size exclusion and ion exchange chromatography. Purified protein concentration was measured by amino acid analysis and the total yield of purified rMOMP is about ~6 mg per liter of cell culture (mean from three preparations).

**Fig. 4:**
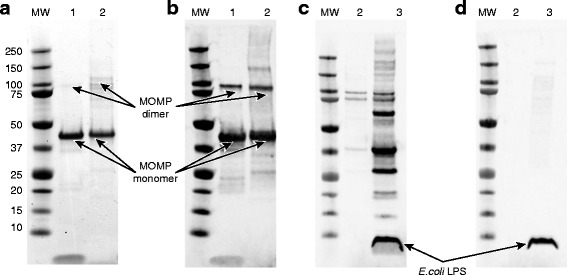
SDS-PAGE and western blot analysis of purified rCtE-MOMP. **a** SDS-PAGE; (**b**) anti-CtE EB mouse sera western blot; (**c**) anti-*E.coli* control nOMV (native outer membrane vesicle from *E. coli* that does not contain recombinant MOMP gene) mouse sera; (**d**) anti-*E.coli* LPS monoclonal antibody. Sample 1, control nCtE-MOMP; sample 2, purified rCtE-MOMP; sample 3, *E. coli* whole cell lysate; all samples were heated and reduced. Monomeric and dimeric forms of MOMP, as well as *E. coli* LPS are indicated

The purified rCtE-MOMP migrates very similarly to the native CtE-MOMP (nCtE-MOMP) that was purified from *Chlamydia* EBs on a SDS-PAGE gel (Fig. 4a and b). It appears that compared to nMOMP, rMOMP has a slightly higher amount of dimeric and oligomeric forms that are SDS-resistant and independent of disulfide reductants (Fig. 4b). A western blot using antisera generated against outer membrane vesicles from the host *E. coli* strain without the recombinant MOMP gene showed that the purified rMOMP contained some co-purified *E. coli* host proteins (Fig. 4c, sample 2), and the purity is estimated to be about ~70 % as determined by mass spectrometry (unpublished observations). We have attempted to improve the purity of the preparation by using different extraction and purification methods, however, were unable to further remove the co-purified host proteins when the rMOMP protein is expressed without an affinity tag. Complete processing of the PelB leader sequence was confirmed by mass spectrometry (unpublished observations). The endotoxin level in the final purified protein sample is undetectable with an anti-*E.coli* lipo-oligosaccharide (LPS) antibody on western blot (Fig. 4d, sample 2).

**Fig. 5:**
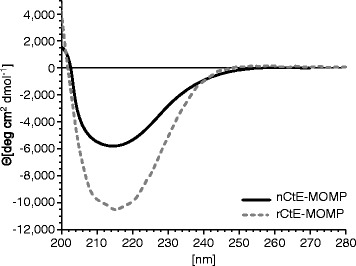
Secondary structure analysis of purified rCtE-MOMP with Circular Dichroism Spectrometry. Mean molar ellipticity was plotted against wavelength. Solid line, purified native CtE-MOMP; dashed line, purified recombinant CtE-MOMP

To investigate whether the recombinantly expressed rCtE-MOMP possessed β-strand secondary structure, we performed circular dichroism (CD) analysis. CD spectra of nMOMP and rMOMP both have a peak at ~215 nm which is the signature of β-strand structure (Fig. 5). Interestingly, rMOMP appears to have a significant higher mean molar ellipticity as compared to the native MOMP. One possible explanation is that the recombinant and native MOMPs were extracted and purified differently, even though both were exchanged into the same final storage buffer. The co-purified *E. coli* host proteins might also contribute to the difference observed.

### Mouse immunogenicity study

Our next goal was to evaluate the immunogenicity of rCtE-MOMP in mice. Female C57BL/6 mice were immunized three times by subcutaneous (s.c.) routes with purified nCtE-MOMP or rCtE-MOMP (10 μg/mouse/immunization) in combination with an adjuvant containing CpG and Montanide on days 0, 20 and 30. A positive control group was immunized with 1x10^6^ live EB per mouse by intraperitoneal (i.p.) route. A negative control group (adjuvant control) was administered with a combination of CpG and Montanide only.

**Fig. 6:**
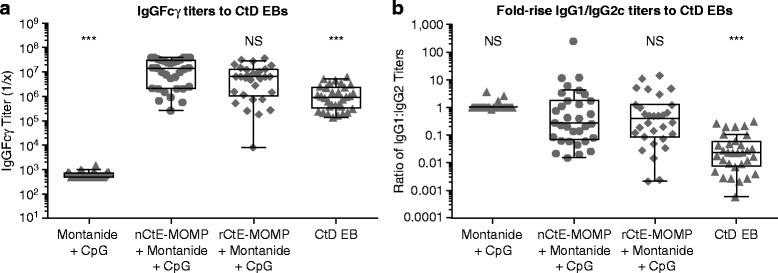
Serum antibody responses against CtD EB in immunized mice. **a** IgG Fcγ antibody titers; (**b**) IgG1/IgG2c ratios. Raw data were plotted on Log10 scale with Box and Whisker Plots (Tukey), boxes = medians with interquartile (IQR) ranges, whiskers = 1.5 times the IQR distances. Transformed data were analyzed by One-way ANOVA with Dunnett post test (compared to CtE nMOMP + Montanide + CpG), * p < 0.05, ** p < 0.01, *** p < 0.001, NS not significant

Post-immunization mouse serum was analyzed by ELISA with CtD EBs as the coating antigen (Fig. 6). The rMOMP immunized mice have comparable (no statistical difference) IgGFcγ titers to the nMOMP immunized group (Fig. 6a). We also tested subtypes of the antibodies to evaluate whether the vaccine responses are Th1 or Th2 biased. rCtE-MOMP elicited similar antibody profiles to the nMOMP immunized group (Fig. 6b). These data suggested that the OM expressed rMOMP elicits a similar antibody response in mice that react to the native *Chlamydia* EB antigen, as compared to the native MOMP.

**Fig. 7:**
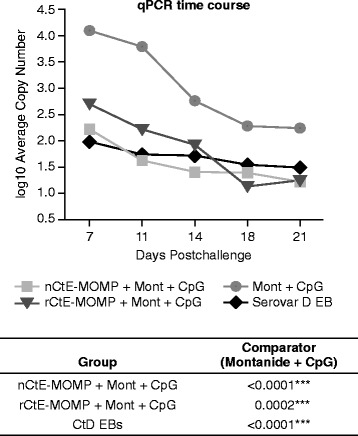
Mouse challenge study. Average copy numbers from real-time PCR for each immunization group were plotted on Log10 scale over the time course. Individual AUC (area under curve) values were calculated for the entire time course and Wilcoxon rank sum was used to determine the p-values for each group (compared to Mont + CpG adjuvant control): * p < 0.05, ** p < 0.01, *** p < 0.001, NS not significant

Two weeks following the last immunization, mice were challenged intravaginally with CtD EBs. The vaginal vault and ectocervix were swabbed on multiple time points (day 7 to day 21) following challenge. Previously, a comparative analysis between a real-time quantitative PCR (qPCR) and inclusion forming unit (IFU) measurement was performed and no major differences between the two assays were observed in monitoring infections [31]. IFU analyis is more labor intensive, more subjective, and not as high-throughput as qPCR. Therefore, qPCR assay was chosen for evaluating our vaccines in this study. rCtE-MOMP elicited significant reduction in *Chlamydial* shedding compared to the adjuvant only control group (P < 0.01) (Fig. 7). Moreover, the amount of shedding after bacterial challenge in mice immunized with rMOMP was comparable (similar bacterial load post-challenge) to the amount observed in the nMOMP immunized group (Fig. 7).

## Discussion

*E. coli* has been the most prominent host system for production of recombinant proteins [29, 32]. However, the *E. coli* cytoplasmic expression system does have some drawbacks. It is not suitable for production of certain proteins, for example, proteins containing complex disulfide bonds. Overexpressed proteins are often produced in the form of inclusion bodies, from which biologically active proteins can only be recovered by complicated denaturation and refolding processes that often have a low recovery. Overexpression of membrane proteins adds additional complexity. We applied a variety of techniques, including the adjustment of codon usage bias, evaluation of different promoters and vectors with different copy numbers to regulate the level of gene transcription, evaluation of different leader sequences to guide the secretion and insertion of the target protein into outer membrane, and evaluation of cell culture medium and optimization of cell culture conditions. In this paper, we demonstrated significant improvement for the recombinant expression of *Chlamydia* MOMP with some of the techniques mentioned above.

Codon usage is a reflection of the relative amounts of cognate aminoacyl tRNAs accessible in the cytoplasm. The synonymous codon usage in all the organisms displays a bias—some codons are used frequently, while others are rarely used. Like others cellular systems, *E. coli* also shows disparities for synonymous codons where in general major codons are predominantly present in highly expressed genes and rare codon occurs in low level expressed genes. This disproportion of codon frequency preferences becomes highly critical for recombinant expression systems, and can lead to mRNA instability, ribosomal stalling, translation errors or premature translational terminations, and ultimately failure to express recombinant genes by heterologous expression hosts. Reported studies indicated that folding and post-translational modifications for both membrane and soluble proteins are also susceptible to synonymous codon usage [33]. Recent studies have shown that codon harmonization, a methodology that matches the codon usage in the recombinant host closely to the usage in the gene’s native organism including the usage of low abundance rare codons (unlike “codon optimization” methodology that often excludes rare codons), can significantly improve the yield and solubility of recombinant proteins [26, 27]. Presumably by this codon selection, pauses in the translation process are incorporated which leads to improved protein folding and efficient protein biosynthesis. In this study, we adapted the concept of codon harmonization and demonstrated successful recombinant expression of the *Chlamydia* MOMP, a cysteine-rich, full-length β-barrel integral membrane protein.

Outer membrane expression of β-barrel proteins in gram-negative bacteria is complicated, involving translation, secretion across inner membrane into the periplasmic space in the unfolded state, simultaneous cognate chaperone assisted folding and insertion into the outer membrane via β-barrel assembly machinery (BAM) complex [34, 35]. Here, we evaluated different promoters, vectors, leader sequences and cell culture conditions for increased outer membrane rMOMP expression. Our data suggested that the combination of a moderate promoter and a low vector copy number greatly improved rMOMP surface expression. We hypothesized that slower transcription and therefore slower translation is optimal for rMOMP OM expression as it provides ample time to allow the newly synthesized protein to be transported into the periplasmic space, properly fold there and translocate onto the outer membrane, resulting in an increased level of surface expression. We have also evaluated different secretion leader sequences, as the efficiency of protein secretion is affected by the leader sequence, even though many prokaryotic and eukaryotic leader sequences are functionally interchangeable. We found that leader sequences originated from the expression host *E. coli*, such as OmpA and OmpP, drove higher levels of rMOMP surface expression as compared to the native *Chlamydia* leader sequences. Surface expression of rMOMP has been associated with toxicity to the host cell [22, 24]. As we need to strike a balance between rMOMP OM expression and the host cell viability, many factors need to be optimized to achieve the best outcome, including optimal cell culture medium, induction of protein expression on or before mid-log phase, and induction at a certain temperature. Our data suggested that the higher surface expression level of a membrane protein such as MOMP can be achieved with a combination of molecular biology and cell culture techniques.

A well-known step along the protein folding pathway that often requires catalysis *in vivo* is the cis/trans isomerization of prolyl-iminopeptide bonds [36]. Four peptidyl-prolyl cis/trans-isomerases (PPIases), SurA, PpiD, FkpA and PpiA, have been described for the isomerization role in periplasm of *E. coli* [37]. We hypothesized that co-expression of these enzymes might improve the efficiency of periplasmic folding of rMOMP and therefore enhance its OM expression level. In addition, proper disulfide bond formation is also presumed to be critical for correct folding of MOMP, a cysteine-rich protein. Both DsbA and DsbC, members of the thioredoxin superfamily, are critical for catalysis of correct disulfide bonds [38]. Another future attempt will be to test the OM expression of rMOMP in an *E. coli* host strain that overexpresses these oxidoreductases.

Refolded rMOMP from *E. coli* inclusion bodies resulted in a reduced level of protection compared to the native MOMP in a mouse challenge model, suggesting that correct conformation of MOMP is required for protective immune responses [23]. Consistent with this finding, rMOMP extracted from inclusion bodies and refolded *in vitro* elicited a significant lower antibody titer against *Chlamydia* EB (unpublished observations). We showed here that OM expressed rMOMP maintained β-strand secondary structure as expected and elicited a comparable antibody response in mice that react to the native *Chlamydia* EB antigen, as compared to the native MOMP. Whether the native tertiary structure is preserved in OM expressed rMOMP remains to be determined, as no molecular structures of either native MOMP or recombinant MOMP are available to date. However, our data suggested that the OM expressed rMOMP at least contains some epitopes in their native forms.

Strains of *Chlamydia trachomatis* human serovars have been used as the challenge strain in murine genital tract models, even though intravaginal inoculation with human serovars typically results in a mild genital tract infection of short duration and lower bacterial burdens [39, 40]. We used *C. trachomatis* serovar D as the challenge strain in our mouse immunogenicity model, and demonstrated that immunization with either native or OM expressed rMOMP resulted in similar low levels of post-challenge bacterial burden, consistent with the comparable antibody responses elicited from rMOMP and nMOMP immunizations. A recent study in adaptive immune deficient mice which lack mature T and B cell immunity but maintain functional innate immune effectors showed that *C. muridarum* infection was unable to be resolved but *C. trachomatis* infection was spontaneously cleared, suggesting a possibility that innate immunity is sufficient to clear the *C. trachomatis* infection in mice [41]. Therefore, the comparison of vaccine efficacy between recombinant and native MOMPs needs to be also evaluated in the more stringent *C. muridarum* mouse challenge model to further confirm the role of adaptive immune response. However, the techniques that we applied to successfully achieve recombinant *Chlamydia* MOMP outer membrane expression can serve as a platform to produce other outer membrane proteins as vaccines or drug candidates and useful reagents for research, as many of these are membrane proteins.

## Conclusions

*C. trachomatis* MOMP is an immunodominant surface protein of crucial importance in the immune response to *Chlamydia* infection and also a major subunit vaccine target. Recombinant MOMP expressed in *E. coli* cytoplasm forms inclusion bodies and rMOMP extracted from inclusion bodies results in a reduced level of protection compared to the native MOMP in a mouse challenge model. Here we demonstrated successful recombinant expression of MOMP on the *E. coli* outer membrane achieved with a variety of techniques, including codon harmonization, utilization of low copy number vectors and promoters with moderate strength, suitable leader sequences and optimization of cell culture conditions. The *E. coli* OM expressed and purified rMOMP is immunogenic in mice and elicits antibodies that react to the native antigen, *Chlamydia* elementary body. Using *C. trachomatis* serovar D as the challenge strain in our mouse immunogenicity model, we demonstrated that immunization with either native or OM expressed rMOMP resulted in similar low levels of post-challenge bacterial burden. Surface expression of rMOMP on *E. coli* OM could provide useful reagents for vaccine research, and the methodology could also serve as a platform to produce other outer membrane proteins.

## References

[1] Fitch WM, Peterson EM, de la Maza LM (1993). Phylogenetic analysis of the outer-membrane-protein genes of Chlamydiae, and its implication for vaccine development. Mol Biol Evol.

[2] Stephens RS, Sanchez-Pescador R, Wagar EA, Inouye C, Urdea MS (1987). Diversity of Chlamydia trachomatis major outer membrane protein genes. J Bacteriol.

[3] Brunham RC, Rey-Ladino J (2005). Immunology of Chlamydia infection: implications for a Chlamydia trachomatis vaccine. Nat Rev Immunol.

[4] Montoya JG. Chlamydia. In: Wilson W, editor. Current Diagnosis & Treatment in Infectious Diseases. New York, NY, USA: The McGraw-Hill Companies, Inc; 2001. p. 694–702.

[5] Grayston JT, Wang S (1975). New knowledge of chlamydiae and the diseases they cause. J Infect Dis.

[6] Schachter J (1978). Chlamydial infections. N Engl J Med.

[7] Washington AE, Katz P (1991). Cost of and payment source for pelvic inflammatory disease. Trends and projections, 1983 through 2000. JAMA.

[8] Stagg AJ (1998). Vaccines against Chlamydia: approaches and progress. Mol Med Today.

[9] Caldwell HD, Kromhout J, Schachter J (1981). Purification and partial characterization of the major outer membrane protein of Chlamydia trachomatis. Infect Immun.

[10] Hatch TP, Vance DW, Al-Hossainy E (1981). Identification of a major envelope protein in Chlamydia spp. J Bacteriol.

[11] Caldwell HD, Perry LJ (1982). Neutralization of Chlamydia trachomatis infectivity with antibodies to the major outer membrane protein. Infect Immun.

[12] Knight SC, Iqball S, Woods C, Stagg A, Ward ME, Tuffrey M (1995). A peptide of Chlamydia trachomatis shown to be a primary T-cell epitope in vitro induces cell-mediated immunity in vivo. Immunology.

[13] Baehr W, Zhang YX, Joseph T, Su H, Nano FE, Everett KD, Caldwell HD (1988). Mapping antigenic domains expressed by Chlamydia trachomatis major outer membrane protein genes. Proc Natl Acad Sci U S A.

[14] Pal S, Theodor I, Peterson EM, de la Maza LM (2001). Immunization with the Chlamydia trachomatis mouse pneumonitis major outer membrane protein can elicit a protective immune response against a genital challenge. Infect Immun.

[15] Pal S, Davis HL, Peterson EM, de la Maza LM (2002). Immunization with the Chlamydia trachomatis mouse pneumonitis major outer membrane protein by use of CpG oligodeoxynucleotides as an adjuvant induces a protective immune response against an intranasal chlamydial challenge. Infect Immun.

[16] Cambridge CD, Singh SR, Waffo AB, Fairley SJ, Dennis VA (2013). Formulation, characterization, and expression of a recombinant MOMP Chlamydia trachomatis DNA vaccine encapsulated in chitosan nanoparticles. Int J Nanomedicine.

[17] Kalbina I, Wallin A, Lindh I, Engström P, Andersson S, Strid K (2011). A novel chimeric MOMP antigen expressed in Escherichia coli, Arabidopsis thaliana, and Daucus carota as a potential Chlamydia trachomatis vaccine candidate. Protein Expr Purif.

[18] Tifrea DF, Ralli-Jain P, Pal S, de la Maza LM (2013). Vaccination with the recombinant major outer membrane protein elicits antibodies to the constant domains and induces cross-serovar protection against intranasal challenge with Chlamydia trachomatis. Infect Immun.

[19] Confer AW, Ayalew S (2013). The OmpA family of proteins: roles in bacterial pathogenesis and immunity. Vet Microbiol.

[20] Rodríguez-Marañón MJ, Bush RM, Peterson EM, Schirmer T, de la Maza LM (2002). Prediction of the membrane-spanning beta-strands of the major outer membrane protein of Chlamydia. Protein Sci.

[21] Wang Y, Berg EA, Feng X, Shen L, Smith T, Costello CE, Zhang YX (2006). Identification of surface-exposed components of MOMP of Chlamydia trachomatis serovar F. Protein Sci.

[22] Hoelzle LE, Hoelzle K, Wittenbrink MM (2003). Expression of the major outer membrane protein (MOMP) of Chlamydophila abortus, Chlamydophila pecorum, and Chlamydia suis in Escherichia coli using an arabinose-inducible plasmid vector. J Vet Med B Infect Dis Vet Public Health.

[23] Sun G, Pal S, Weiland J, Peterson EM, de la Maza LM (2009). Protection against an intranasal challenge by vaccines formulated with native and recombinant preparations of the Chlamydia trachomatis major outer membrane protein. Vaccine.

[24] Koehler JE, Birkelund S, Stephens RS (1992). Overexpression and surface localization of the Chlamydia trachomatis major outer membrane protein in Escherichia coli. Mol Microbiol.

[25] Findlay HE, McClafferty H, Ashley RH (2005). Surface expression, single-channel analysis and membrane topology of recombinant Chlamydia trachomatis Major Outer Membrane Protein. BMC Microbiol.

[26] Angov E, Hillier CJ, Kincaid RL, Lyon JA (2008). Heterologous protein expression is enhanced by harmonizing the codon usage frequencies of the target gene with those of the expression host. PLoS One.

[27] Angov E (2011). Codon usage: nature’s roadmap to expression and folding of proteins. Biotechnol J.

[28] Pal S, Theodor I, Peterson EM, de la Maza LM (1997). Immunization with an acellular vaccine consisting of the outer membrane complex of Chlamydia trachomatis induces protection against a genital challenge. Infect Immun.

[29] Makrides SC (1996). Strategies for achieving high-level expression of genes in Escherichia coli. Microbiol Rev.

[30] Robert V, Volokhina EB, Senf F, Bos MP, Van Gelder P, Tommassen J (2006). Assembly factor Omp85 recognizes its outer membrane protein substrates by a species-specific C-terminal motif. PLoS Biol.

[31] Wooters MA, Kaufhold RM, Field JA, Indrawati L, Heinrichs JH, Smith JG (2009). A real-time quantitative polymerase chain reaction assay for the detection of Chlamydia in the mouse genital tract model. Diagn Microbiol Infect Dis.

[32] Lee SY (1996). High cell-density culture of Escherichia coli. Trends Biotechnol.

[33] Gustafsson C, Govindarajan S, Minshull J (2004). Codon bias and heterologous protein expression. Trends Biotechnol.

[34] Knowles TJ, Scott-Tucker A, Overduin M, Henderson IR (2009). Membrane protein architects: the role of the BAM complex in outer membrane protein assembly. Nat Rev Microbiol.

[35] Hagan CL, Kim S, Kahne D (2010). Reconstitution of outer membrane protein assembly from purified components. Science.

[36] Fischer G, Tradler T, Zarnt T (1998). The mode of action of peptidyl prolyl cis/trans isomerases in vivo: binding vs. catalysis. FEBS Lett.

[37] Baneyx F, Mujacic M (2004). Recombinant protein folding and misfolding in Escherichia coli. Nat Biotechnol.

[38] Bardwell JC, McGovern K, Beckwith J (1991). Identification of a protein required for disulfide bond formation in vivo. Cell.

[39] Morrison RP, Caldwell HD (2002). Immunity to murine chlamydial genital infection. Infect Immun.

[40] Perry LL, Su H, Feilzer K, Messer R, Hughes S, Whitmire W, Caldwell HD (1999). Differential sensitivity of distinct Chlamydia trachomatis isolates to IFN-gamma-mediated inhibition. J Immunol.

[41] Sturdevant GL, Caldwell HD (2014). Innate immunity is sufficient for the clearance of Chlamydia trachomatis from the female mouse genital tract. Pathog Dis.

